# Resistance trends in gram-negative bacteria: surveillance results from two Mexican hospitals, 2005–2010

**DOI:** 10.1186/1756-0500-5-277

**Published:** 2012-06-07

**Authors:** Rayo Morfin-Otero, Juan Carlos Tinoco-Favila, Helio S Sader, Lorena Salcido-Gutierrez, Hector Raul Perez-Gomez, Esteban Gonzalez-Diaz, Luis Petersen, Eduardo Rodriguez-Noriega

**Affiliations:** 1 Instituto de Patología Infecciosa y Experimental, Centro Universitario Ciencias de la Salud, Universidad de Guadalajara, Calle Hospital 308, Colonia El Retiro, CP 44280, Guadalajara, Jalisco, Mexico; 2 Infectología, Microbiología, Hospital Civil de Guadalajara, Fray Antonio Alcalde, Guadalajara, Mexico; 3 Laboratorio de Microbiología, Hospital General de Durango, Secretaria de Salud, Durango, Mexico; 4 JMI Laboratories, North Liberty, IA, USA

**Keywords:** Bacterial, Resistance, Gram negative, Nosocomial, Infections

## Abstract

**Background:**

Hospital-acquired infections caused by multiresistant gram-negative bacteria are difficult to treat and cause high rates of morbidity and mortality. The analysis of antimicrobial resistance trends of gram-negative pathogens isolated from hospital-acquired infections is important for the development of antimicrobial stewardship programs. The information obtained from antimicrobial resistant programs from two hospitals from Mexico will be helpful in the selection of empiric therapy for hospital-acquired gram-negative infections.

**Findings:**

Two thousand one hundred thirty two gram-negative bacteria collected between January 2005 and December 2010 from hospital-acquired infections occurring in two teaching hospitals in Mexico were evaluated. *Escherichia coli* was the most frequently isolated gram-negative bacteria, with >50% of strains resistant to ciprofloxacin and levofloxacin. *Klebsiella* spp. showed resistance rates similar to *Escherichia coli* for ceftazidime (33.1% vs 33.2%), but exhibited lower rates for levofloxacin (18.2% vs 56%). Of the samples collected for the third most common gram-negative bacteria, *Pseudomonas aeruginosa*, >12.8% were resistant to the carbapenems, imipenem and meropenem. The highest overall resistance was found in *Acinetobacter* spp. *Enterobacter* spp. showed high susceptibility to carbapenems.

**Conclusions:**

*E. coli* was the most common nosocomial gram-negative bacilli isolated in this study and was found to have the second-highest resistance to fluoroquinolones (>57.9%, after *Acinetobacter* spp. 81.2%). This finding represents a disturbing development in a common nosocomial and community pathogen.

## Findings

### Rational for the surveillance of bacterial resistance trends

Gram-negative infections are responsible for a large portion of device-associated infections, procedure-associated infections, and healthcare-associated infections [1]. Recent data from the National Healthcare Safety Network indicate that gram-negative bacteria are responsible for more than 30% of hospital-acquired infections and more than 40% of infections in patients in intensive care units [2,3]. Hospital-acquired infections caused by gram-negative bacteria are difficult to manage, due to the increasingly varied resistance mechanisms that these bacteria can develop [4,5].The continuous surveillance of antibiotic resistance trends in bacteria isolated from hospital-acquired infections is essential for the selection of adequate initial empiric therapy [6,7].The laboratory-based antibiograms is efficacious as a guide for the rational selection of antimicrobial therapy, and to alert healthcare providers to the presence of unusual or emerging antimicrobial mechanisms [8]. The evaluation of antimicrobial resistance in gram-negative bacterial strains in two Mexican hospitals during 2005–2010 is presented.

## Methods

The participating hospitals in this study are similar in their patient characteristics. The Hospital Civil de Guadalajara Fray Antonio Alcalde is a 1,000 bed tertiary care teaching hospital located in the city of Guadalajara, the second largest city in Mexico. The Hospital General de Durango is a 300-bed teaching hospital located in the city of Durango, which is the capital of the state of Durango in Mexico.

All isolates were identified at the participating institution by routine methodologies that are in use at each laboratory. Upon receipt at the central monitor (JMI Laboratories, North Liberty, IA, USA), isolates were subcultured to ensure viability and purity. Confirmation of species identification was performed with the Vitek system (bioMérieux Vitek, St Louis, MO) [9,10].

A total of 2132 gram-negative bacteria were collected between January 2005 and December 2010 and were analyzed in the present study. The organisms were consecutively collected according to the types of infection, which primarily included bloodstream infections, skin and skin structure infections, and pneumonia in hospitalized patients. The organisms evaluated in this study included *E. coli* (563 strains), *Klebsiella* spp. (329 strains), *P. aeruginosa* (404 strains), *Acinetobacter* spp. (362 strains) and *Enterobacter* spp. (214 strains).

Included among 260 other gram-negatives collected were *Citrobacter* spp. (32 strains, including 26 *Citrobacter freundii*), *Proteus* spp. (34 strains, including 29 *Proteus mirabilis*), *Serratia* spp. (64 strains, including 61 *Serratia marcescens*), *Stenotrophomonas maltophilia* (37 strains), *Pseudomonas fluorescens* (10 strains), *Salmonella* spp. (24 strains, including 2 *Salmonella cholerasuis*, 1 *Salmonella paratyphi*), and 59 (<3 isolates) other gram-negatives.

Antimicrobial susceptibility testing was performed using the broth microdilution method following the recommendations of the Clinical and Laboratory Standards Institute, M07-A8 [11].Antimicrobial powders were obtained from the respective manufacturers, and microdilution plates were prepared by ThermoFisher Scientific (formerly TREK Diagnostics; Cleveland, OH, USA). The susceptibility results were interpreted according to the Clinical Laboratory Standards Institute document M100-S21 [12-16].

*E. coli* and *Klebsiella pneumoniae* isolates with MIC values of ≥ 2 μg/mL for aztreonam and/or ceftazidime and/or ceftriaxone were considered extended spectrum betalactamases (ESBL) phenotypes[17,18]. Quality control was established by testing *E. coli* ATCC 25922, *P. aeruginosa* ATCC 27853, *Staphylococcus aureus* ATCC 29213, and *Streptococcus pneumoniae* ATCC 49619.

Linear trend analysis for resistance trend over time was performed using SPSS statistical software, version 17.0.

## Results

The most common gram-negative isolate was *E. coli* (Table 1). Of the *E. coli strains*, 33.2% were resistant to ceftazidime; >55% were resistant to the two fluoroquinolones tested, ciprofloxacin and levofloxacin; and 31.9% were resistant to gentamicin (Table 1). The *E. coli* isolates were consistently susceptible to carbapenems and amikacin, (100.0% and 95.7%, respectively), while piperacillin/tazobactam was active against 83.1% of strains at the susceptible breakpoint (Table 1).

**Table 1:** **Comparison of the in vitro activities of selected antimicrobial agents tested against*Escherichia coli*(563 strains)**

Antimicrobial agent	MIC_50_	MIC_90_	Range	% susceptible/resistant^a^
Cefuroxime	8	>16	≤2 – >16	55.2 / 43.2
Cefoxitin	4	>16	≤4 – >16	74.9 / 13.7
Ceftriaxone	≤0.25	>32	≤0.25 – >32	56.8 / 41.7
Ceftazidime	≤1	>16	≤1 – >16	61.6 / 33.2
Cefepime	0.25	>16	≤0.12 – >16	71.4 / 19.9
Piperacillin/tazobactam	4	32	≤0.5 – >64	83.1 / 3.9
Imipenem	≤0.12	0.25	≤0.12 – 1	100.0 / 0.0
Meropenem	≤0.12	≤0.12	≤0.12 – 0.5	100.0 / 0.0
Ciprofloxacin	>4	>4	≤0.03 – >4	41.7 / 57.9
Levofloxacin	>4	>4	≤0.5 – >4	41.7 / 56.7
Gentamicin	≤2	>8	≤2 – >8	67.1 / 31.9
Amikacin	≤4	8	≤4 – >32	95.7 / 0.4
PolymyxinB^b^	≤0.5	≤0.5	≤0.5 – 1	100 / 0.0
Colistin^b^	≤0.5	≤0.5	≤0.5 – 2	99.8 / 0.2

^a^ Criteria as published by the CLSI [16].

^b^*Pseudomonas aeruginosa* breakpoints.

*Klebsiella* spp. showed high resistant rates to ceftazidime (33.1% compared to 24.0% in *P. aeruginosa*), but relatively low resistance to fluoroquinolones (≤18.2% vs. >50% in *E. coli*), more resistant to amikacin (13.1% vs. 0.4% in *E. coli*), and had similar susceptibility rates to the carbapenems as *E. coli*, ≥ 98.4% (Table 2).

**Table 2:** **Comparison of the in vitro activities of selected antimicrobial agents tested against*Klebsiella*spp.**^**a**^**(329 strains)**

Antimicrobial agent	MIC_50_	MIC_90_	Range	% susceptible/resistant^b^
Cefuroxime	4	>16	≤2 – >16	62.3 / 32.2
Cefoxitin	≤4	>16	≤4 – >16	82.6 / 11.8
Ceftriaxone	≤0.25	32	≤0.25 – >32	63.8 / 35.2
Ceftazidime	≤1	>16	≤1 – >16	66.8 / 33.1
Cefepime	≤0.12	8	≤0.12 – >16	92.7 / 4.5
Piperacillin/tazobactam	2	>64	≤0.5 – >64	79.3 / 11.2
Imipenem	0.25	0.5	≤0.12 – >8	98.4 / 1.2
Meropenem	≤0.12	0.25	≤0.12 – >8	98.4 / 0.9
Ciprofloxacin	≤0.03	>4	≤0.03 – >4	80.8 / 18.2
Levofloxacin	≤0.5	>4	≤0.5 – >4	82.3 / 15.8
Gentamicin	≤2	>8	≤2 – >8	82.6 / 14.5
Amikacin	2	>32	≤4 – >32	84.1 / 13.1
PolymyxinB	≤0.5	≤0.5	≤0.5 – >4	99.2 / 0.6
Colistin	≤0.5	≤0.5	≤0.5 – >4	99.5 / 0.4

^a^ Includes: Klebsiellaoxytoca (36 strains) Klebsiellapneumoniae (291 strains), Klebsiellaornithinolytica (1 strain) and unspeciated Klebsiella (1 strain).

^b^ Criteria as published by the CLSI [16].

^c^*Pseudomonas aeruginosa* breakpoints.

Of the isolated gram-negative bacteria, *Pseudomonas aeruginosa* was the third most common organism after *E. coli* and *Klebsiella* spp. (Table 3). *P. aeruginosa* exhibited high resistance rates to the two carbapenems tested, 17.8% of the isolates were resistant to imipemen and 12.8% were resistant to meropenem (Table 3).

**Table 3:** **Comparison of the in vitro activities of selected antimicrobial agents tested against*Pseudomonas aeruginosa*(404 strains)**

Antimicrobial agent	MIC_50_	MIC_90_	Range	% susceptible / resistant^a^
Ceftazidime	2	>16	≤1 – >16	71.7 / 24.0
Cefepime	4	16	0.5 – >16	77.9 / 9.9
Piperacillin/tazobactam^b^	8	>64	≤0.5 – >64	68.8 / 15.5
Imipenem	2	>8	≤0.12 – >8	74.9 / 17.8
Meropenem	0.5	>8	≤0.12 – >8	76.4 / 12.8
Ciprofloxacin	0.25	>4	≤0.03 – >4	75.2 / 20.0
Levofloxacin	≤0.5	>4	≤0.5 – >4	74.2 / 24.0
Gentamicin	≤2	>8	≤2 – >8	64.1 / 31.9
Amikacin	4	>32	≤4 – >32	70.3 / 23.7
PolymyxinB	1	1	≤0.5 – 2	100.0 / 0.0
Colistin	1	2	≤0.5 – 2	100.0 / 0.0

^a^ Criteria as published by the CLSI [16].

^b^ Criteria published by the CLSI [15].

*Acinetobacter* spp, the fourth most common gram-negative bacilli isolated during this study, was the most resistant to the antimicrobials tested (Table 4). More than 60% of the *Acinetobacter* spp. isolates were resistant to all antibiotics tested, except imipenem (36.4% resistance), meropenem (37.4% resistance) and colistin / polymyxin B, 1.5 / 1.4% resistance (Table 4).

**Table 4:** **Comparison of the in vitro activities of selected antimicrobial agents tested against*Acinetobacte*spp.**^**a**^**(362 strains)**

Antimicrobial agent	MIC_50_	MIC_90_	Range	% susceptible / resistant^b^
Ceftazidime	>16	>32	≤1 – >16	17.1 / 74.3
Cefepime	16	>16	≤0.12 – >16	28.7 / 49.4
Piperacillin / tazobactam	>64	>64	≤0.5 – >64	16.3 / 77.9
Imipenem	4	>8	≤0.12 – >8	52.3 / 36.4
Meropenem	4	>8	≤0.12 – >8	52.3 / 37.4
Ciprofloxacin	>4	>4	≤0.03 – >4	18.2 / 81.2
Levofloxacin	>4	>4	≤0.5 – >4	18.7 / 78.1
Gentamicin	>8	>8	≤2 – >8	34.2 / 63.5
Amikacin	>32	>32	≤4 – >32	22.1 / 64.9
PolymyxinB	≤0.5	≤0.5	≤0.5 – >4	98.6 / 1.4
Colistin	≤0.5	1	≤0.5 – >4	98.5 / 1.5

^a^ Includes *Acinetobacterbaumannii* (295 strains), *Acinetobacterhaemolyticus* (3 strains), *Acinetobacterlwoffii* (16 strains), and unspeciated *Acinetobacter* (48 strains).

^b^ Criteria as published by the CLSI [16].

*Enterobacter* spp., the fifth most frequently isolated gram-negative bacilli, had a different resistance pattern than the other gram-negative bacilli tested (Table 5). All (100.0%) *Enterobacter* spp. tested were susceptible to imipenem and meropenem. Only 3.7% were resistant to cefepime, 26.1% were resistant to piperacillin/tazobactam, 14.0% were resistant to ciprofloxacin, and 12.6% were resistant to levofloxacin (Table 5).

**Table 5:** **Comparison of the in vitro activities of selected antimicrobial agents tested against*Enterobacterspp*.**^**a**^**(214 strains)**

Antimicrobial agent	MIC_50_	MIC_90_	Range	%susceptible / resistant^b^
Ceftriaxone	0.25	>32	≤0.25 – >32	59.3 / 39.2
Ceftazidime	≤1	>16	≤1 – >16	62.6 / 34.5
Cefepime	≤0.12	8	≤0.12 – >16	92.1 / 3.7
Piperacillin / tazobactam	2	>64	≤0.5 – >64	73.8 / 26.1
Imipenem	0.5	1	≤0.12 – 8	98.6 / 0.0
Meropenem	≤0.12	0.12	≤0.12 – 4	100.0 / 0.0
Ciprofloxacin	≤0.03	>4	≤0.03 – >4	85.0 / 14.0
Levofloxacin	≤0.5	>4	≤0.5 – >4	87.4 / 12.6
Gentamicin	≤2	>8	≤2 – >8	81.3 / 18.6
Amikacin	2	>32	≤4 – >32	82.7 / 16.3
PolymyxinB	≤0.5	>4	≤0.5 – >4	- / -
Colistin	≤0.5	>4	≤0.5 – >4	- / -

^a^ Includes *Enterobacteraerogenes* (38 strains), *Enterobacteramnigenus* (2 strains), *Enterobactercancerogenus* (1 strain), *Enterobacter cloacae* (161 strains), *Enterobactergergoviae* (6 strains), *Enterobactersakazakii* (4 strains), and unspeciated *Enterobacter* (2 strains).

^b^ Criteria as published by the CLSI [16].

During the observation period *E. coli* with an ESBL phenotype increased from 35.0% in 2005 to 52.4% in 2010(p < 0.008), *Klebsiella* spp. with an ESBL phenotype increased from 40.5% in 2005 to 43.8% in 2010, imipenem-non-susceptible *Klebsiella* spp.phenotype decreased from 8.1% in 2005 to 2.1% in 2010, ceftazidime-resistant *Enterobacter* spp.phenotype increased from 32.7% in 2005 to 46.4% in 2010, imipenem-non-susceptible *Enterobacter* spp. phenotype increased from 2.0% in 2005 to 3.6% in 2010, imipenem-resistant *Acinetobacter* spp. phenotype increased from 13.8% in 2005 to 63.5% in 2010 (p < 0.001), and the imipenem-resistant *P. aeruginosa* phenotype increased from 16.8% in 2005 to 22.1% in 2010 (Table 6).

**Table 6:** Yearly variation of main resistance phenotypes

Resistance phenotype	Year of isolation (Total/Percentage)					
	2005	2006	2007	2008	2009	2010
*E. coli* ESBL phenotype^a,f^	36 (35.0)	26(37.7)	29(38.7)	42(40.0)	45(54.2)	67(52.4)
*Klebsiella* spp. ESBL phenotype^a^	37(40.5)	17(33.3)	27(41.5)	7.8(20.0)	15(36.6)	21(43.8)
Imipenem-NS*Klebsiella*^b^	7(8.1)	2(2.0)	3(4.6)	0.00	2(4.9)	1(2.1)
Ceztazidime-R *Enterobacter*^c^	16(32.7)	6(16.7)	14(34.1)	10(37.0)	17(47.2)	13(46.4)
Imipenem-NS – *Enterobacter*^d^	1(2.0)	0.00	2(4.5)	4(14.8)	2(5.6)	1(3.6)
Imipenem-R *Acinetobacter*^e,f^	4(13.8)	3(8.8)	6(20.0)	40(48.9)	33(65.6)	54(63.5)
Imipenem-R *P. aeruginosa*^e^	13(16.8)	26(32.1)	13(27.1)	21(27.3)	13(25.0)	16(22.1)

^a^ Defined as MIC ≥2 μg/ml for ceftazidime or ceftriaxone or aztreonam [CLSI, 2011].

^b^ Imipenem MIC of ≥2 μg/ml [CLSI, 2011].

^c^ Ceftazidime MIC of ≥16 μg/ml [CLSI, 2011].

^d^ Imipenem MIC of ≥2 μg/ml [CLSI, 2011].

^e^ Imipenem MIC of ≥8 μg/ml [CLSI, 2011].

^f^ Resistance trend over time p<0.05.

### Summary and implications

Overall the resistance pattern found in our analysis in *K. pneumoniaeP. aeruginosaAcinetobacter* spp., and *Enterobacter* spp. is similar to that described in other Mexican and Latin American studies[19-24].

The similar susceptibility to ceftazidime and ceftriaxone in *E.coli* and *Klebsiella* spp. suggests that CTX-M-beta-lactamases are present in our hospitals although not as widely disseminated as it occurred in the United States of America where susceptibility to ceftriaxone is much lower when compared to ceftazidime [25]. The production of CTX-M-type beta-lactamases in association with the production of other extended-spectrum-beta-lactamases have been reported in other areas in Mexico [19,20]. Certain resistant phenotypes encountered in this study are to be examined carefully, including the ESBL phenotype increase in *E. coli*, and the imipenem resistant phenotype increase in *Acinetobacter* spp.

The emergence of resistance to carbapenems and the lack of options for the treatment of *P. aeruginosa* infections with the exception of colistin and polymyxin B are considerable [25,26].

Some of the limitations of our report include the lack of resistance genotyping and of molecular strain typing.

The surveillance data presented by this study will help to guide clinicians in our hospitals in the selection of appropriate empiric antimicrobial treatment when confronted with gram-negative infections. Our findings can be used to monitor the evolution of bacterial resistance in other similar hospitals and will be helpful for the development of antibiotic stewardship programs.

## Competing interests

The authors declare that they have no competing interests.

## Authors’ contributions

Conceived and designed the experiments: HSS, RMO, ERN, JCT. Performed the experiments: HSS, RMO, JCT, LSG, ERN. Acquisition and Analyzed the data: HSS, RMO, JCT, LSG, ERN, HRP, EGD, LP. Contributed reagents/materials/analysis tools: HSS, RMO, JCT, LSG, ERN, HRP, EGD, LP. Wrote the paper: RMO, HSS, JCT, ERN. All authors read and approved the manuscript.
